# Safety reporting of Essure medical device: a qualitative and quantitative assessment on the FDA manufacturer and user facility device experience database in 2018

**DOI:** 10.3389/frph.2023.1172927

**Published:** 2023-07-13

**Authors:** Chenyu Zou, Brandy Davis, Patricia R. Wigle, Ana L. Hincapie, Jeff Jianfei Guo

**Affiliations:** ^1^ Department of Health Outcomes Research and Policy, Harrison College of Pharmacy, Auburn University, Auburn, AL, United States; ^2^ Division of Pharmacy Practice & Administrative Sciences, The James L. Winkle College of Pharmacy, University of Cincinnati, Cincinnati, OH, United States

**Keywords:** adverse event reporting, device safety, Essure, maude, surveillance

## Abstract

**Background:**

There have been numerous cases of adverse events since the introduction of Essure medical devices for sterilization in 2002. This study analyzed the safety event reports of the Essure reported in the Manufacturer and User Facility Device Experience (MAUDE).

**Methods:**

A retrospective analysis examined the MAUDE reports between Jan-1, 2018, and Oct-31, 2018 and focused on safety reports related to the Essure device. Safety reports were categorized and analyzed by their event type, device problem, patients’ symptoms and the level of harm. Of this study cohort, 10% of samples were randomly selected for quantitative analyses. Thematic analysis was conducted for reports included death cases.

**Results:**

A total of 4,994 eligible reports were analyzed. There were ten reports associated with individuals’ deaths, and the main themes of safety reports from qualitative analysis were pains, bleeding, surgery, migraine, and infection. Quantitative analysis of 500 randomly selected samples showed that 98% of adverse event reports were associated with different injuries such as surgery, pain, bleeding, hysterectomy, and menorrhagia. Additionally, more than 90% of reports were submitted by the manufacturer.

**Conclusion:**

These findings indicated several safety issues of Essure. More meaningful pre- and post-marketing surveillance and regulation are warranted in the medical device market to ensure safety and effectiveness, including investigating complaints, promptly sharing relevant information with regulators and users, and implementing corrective actions

## 1. Introduction

The Essure device, produced by Bayer, is a nonhormonal permanent birth control device and does not require general anesthesia with implantation (1, 2). It consists of two coils, an outer coil made of stainless steel and an inner coil made of a nickel titanium alloy. The coils are placed in the fallopian tubes, ultimately resulting in tubal occlusion (3). The implantation procedure of Essure involves no incision and may be completed in ten minutes (2, 4, 5). Until 2013, more than 750,000 Essure procedures were performed worldwide (6–8). However, tens of thousands of women worldwide have suffered from adverse events associated with Essure (9, 10). Reported adverse events include persistent pain, perforation of the uterus and fallopian tubes, intra-abdominal or pelvic device migration, extra bleeding, and hypersensitivity reactions (5, 6, 9–12). Some women needed the device surgically removed, and unintended pregnancies due to Essure’s failure are detailed in case reports (4, 11, 12).

### 1.1. Previous research about Essure safety problems

The first case of Essure tubal sterilization was conducted in Australia in 1999, then the use of Essure spread to the U.S. and Europe (13). The pilot study conducted in 2001 reported no pregnancies, concluding that the Essure contraceptive method was safe and highly recommended for women seeking permanent birth control (14). Nonetheless, some sporadic cases were reported by the literature, and the first publication concerning unwanted pregnancies was published by Levy et al. in 2007 (15). A study in 2015 found a 10-fold increased potential risk of re-operation in the first year for patients with Essure compared with patients who underwent laparoscopic sterilization (16).

An uncommon but serious side effect of Essure is a nickel allergy, and the manufacturer claimed that 0.004% of Essure’s users are likely to have hypersensitivity reactions to nickel (17). As of 2018, only four previous case reports worldwide have suggested that nickel may cause allergic contact dermatitis. For the fourth case, the allergic symptoms were completely resolved after a hysterectomy (18).

### 1.2. Policy implication

Bayer was required by the U.S. Food and Drug Administration (FDA) to add a new boxed warning in 2016 and was also ordered to carry out a post-marketing surveillance study comparing the adverse effects of the Essure and tubal ligation (7, 19, 20). In February 2016, the FDA ordered Bayer to conduct a post-market safety study to help the FDA better understand the risks of Essure comparing with laparoscopic tubal ligation (7, 21). On October 31, 2016, the FDA issued the final guidance including a warning box of safety statement and a checklist for the decision making of permanent birth control choices.

Bayer announced that they would continue to implement FDA restrictions on Essure sales and distribution in the beginning of April 2018. On December 31, 2018, Bayer stopped selling or distributing the Essure devices in the U.S., but Essure could be implanted within one year after purchase (22, 23). As Bayer stated, the reason for their decision was that “the demand for Essure has fallen sharply in many markets recently, and this trend is not expected to change” (8, 23). In December 2018, the FDA approved a revised protocol to extend Bayer’s mandatory follow-up study with continued enrollment of participants (8).

Due to the safety issues Essure has seen, additional and meaningful safeguards are required to ensure women can make informed decisions about potential risks and adverse events (9, 10, 24). The general goal of this study was to describe, review, and analyze the safety event reports about the medical device Essure during its last year in the U.S. market. For women who still have the Essure implant, it is still important to evaluate Essure safety reports and advocate for more effective surveillance of medical devices.

## 2. Material and methods

### 2.1. Data source

The FDA Manufacturer and User Facility Device Experience (MAUDE) database was used to retrieve statistics and information concerning the adverse events of the Essure device. MAUDE is an online public database that includes the medical device adverse reports submitted to the FDA by consumers, health professionals, manufacturers, and device user providers. It is a mandatory requirement for device manufacturers, importers, distributors, and user facilities to report device-related death or serious injury to the FDA (25, 26). MAUDE data are collected from both mandatory report (MedWatch form FDA 3,500A form for user facilities, importers, distributors, and manufacturers) and the voluntary report (MedWatch form FDA 3,500 form for healthcare professionals, consumers, and patients) (27). Approval from the University of Cincinnati’s Institutional Review Board was not required for this study since all data was de-identified and collected from a publicly accessible FDA MAUDE database and previously published research (28).

### 2.2. Target population, sample size and study period

The study samples were all patient safety incidents reported to the FDA MAUDE database in the U.S. from January 1, 2018, to October 31, 2018, which was the most recent publicly available data for medical device safety surveillance. For eligible reports, the device brand name had to include “Essure”, and the manufacturer name had to include “Bayer”. The absence of event text resulted in the exclusion of qualitative analysis due to extremely limited information. According to the “10% condition” in statistics, ten percent of randomized reports were finally included in the quantitative review of this study. The “10% condition” is a guideline used to ensure that samples are not too large relative to the population from which they are drawn. Specifically, the rule states that sample sizes should be no more than 10% of the population size (29, 30). Reports which contained death outcomes were included for the thematic analysis. Eligible records were selected by one reviewer independently.

### 2.3. Data analysis

All included records were analyzed for Report Source, Reporter Occupation, Initial Report to FDA, and Event Type using SAS® 9.4. The word frequency of all eligible reports was calculated using descriptive statistical software NVivo (12 PRO). Considering the different severities of events, the FDA’s definition of serious adverse drug events was used to identify the patients’ outcomes mentioned in the report. A random sample of 10% of all the reports was selected to be reviewed to better understand the safety reports, especially the description of different types of pain.

## 3. Results

A total of 4,994 records were selected for full-text review. Five hundred randomized reports were included in the quantitative review and ten studies containing death outcomes were included in the case studies.

### 3.1. Report characteristics

Characteristics of the reports included are presented in Table 1. The manufacturer submitted 4,509 (90.26%) reports, and “Non-Healthcare Professional” was listed as the occupation in 451 (9.69%) reports. Only 38 reports (0.82%) were initially reported to the FDA. The event type of most reports (98.70%) was “Injury”, and ten (0.20%) reports included “death” outcomes. Partial contents from some reports were missing, including the event date, event type, device problem, and event text. Some reports contained two or more device problems; for example, some samples reported biocompatibility and the device appears to trigger rejection. According to the FDA Product Classification guideline, the product code “HHS” represents a class III, implanted, trans-cervical contraceptive tubal occlusion device.

**Table 1:** Characteristics of included reports.

Characteristics	Full cohort (*n* = 4,994)
Report source (*n*, %)	
Voluntary report	482 (9.65)
User facility report	3 (0.06)
Manufacturer report	4,509 (90.29)
Reporter occupation (*n*, %), frequency missing = 341	
Other	4,195 (90.16)
Physician	3 (0.06)
Non-healthcare professional	451 (9.69)
Risk manager	2 (0.04)
Unknown	2 (0.04)
Initial report to FDA (*n*, %), frequency missing = 342	
Yes	38 (0.82)
No	4,038 (86.80)
Unknown	576 (12.38)
Event type (*n*, %)	
Death	10 (0.20)
Injury	4,929 (98.7)
Malfunction	53 (1.06)
Other	2 (0.04)

### 3.2. Safety event category using qualitative analysis

Table 2 shows the result when the word frequency analysis was narrowed down to words related to particular adverse events, including ten most common words related to adverse events, and ten words related to serious adverse events. The most frequent word about adverse events was “pain”, followed by “hemorrhage”, “surgery”, “migraine”, and “infection”. For serious adverse events, “surgery” is the most frequent word. It is worth noting that although the term “death” was mentioned 5,558 times, it is mostly used to indicate that “this report does not include deaths”, or “death is not related to the device”, which are not related to actual death outcomes.

**Table 2:** Associated with Essure reported in the FDA MAUDE database in 2018 (*N* = 4,994) using nVivo® software.

Word	Count	Related words
Top 10 frequently reported adverse events associated with Essure		
Pain	41,564	Afflictions, anguish, awful, bother, bothering, distress, dreadful, hurt, hurtful, hurting, hurts, irritability, irritable, irritate, irritated, irritates, irritating, irritation, irritations, pain, painful, paining, pains, sore, soreness, sores, strain, terrible, terribly, trouble, troubled
Hemorrhage	14,620	Bleed, bleeding, bleedings, bleeds, haemorrhage, haemorrhagic, haemorrhaging, hemorrhage, hemorrhaged, hemorrhages, hemorrhagic, hemorrhaging
Surgery	6,328	Operated, operating, operation, operations, operative, operatively, surgeries, surgery
Menorrhagia	6,321	Hypermenorrhea, menorrhagia
Migraine	5,207	Migraine, migraines
Infection	5,147	Infected, infection, infections, infectious, infective, septic, transmission
Dyspareunia	4,663	Dyspareunia
Dysmenorrhoea	3,634	Dysmenorrhoea
Perforation	3,033	Penetrated, penetrating, penetration, perforate, perforated, perforating, perforation, perforations, pierced, piercing, piercings, punch, puncture, punctured, punctures, puncturing
Anxiety	3,083	Anxiety, anxious
Top 10 Serious adverse events associated with Essure		
Surgery	6,328	Operated, operating, operation, operations, operative, operatively, surgeries, surgery
Death	5,558	Dead, deadly, death, deaths, decease, deceased, demise, destruction, die, died, dying, end, ended, ending, ends, expiry, last, lasted, lasting, lastly, lasts, mortal
Perforation	3,033	Penetrated, penetrating, penetration, perforate, perforated, perforating, perforation, perforations, pierced, piercing, piercings, punch, puncture, punctured, punctures, puncturing
Hysterectomy	2,373	Hysterectomies, hysterectomy
Cyst	1,780	Cyst, cysts, vesicle, vesicles
Salpingectomy	1,501	Salpingectomies, salpingectomy
Endometriosis	988	Adenomyosis, endometriosis
Anaemia	720	Anaemia, anemia
Hospitalization	469	Hospital, hospitalisation, hospitalization, hospitalizations, hospitalized, hospitals
Incontinence	351	Dissolution, incontinence, incontinency

### 3.3. Quantitative analysis of randomly selected 500 samples

A total of 500 samples (10% of total reported adverse events) were randomly selected for quantitative analysis using the random numbers generator in Excel. One hundred ninety-nine (40%) of the 500 reports contain information about the event date. There were 265 (53%) adverse events that occurred in the past 5 years (2014–2018). All 500 samples reported event types, including eight cases of malfunction (1.6%), one case (0.2%) of death, and 491 cases (98%) of injury. 187 (34%) reports did not identify the device or use problems, and 24 (4.8%) reported biocompatibility (Supplementary Table S1). There were 429 (86%) patients who required external intervention, and 355 (71%) patients who had surgical removal of the Essure device. In a few cases, Essure caused hospitalization (15, 3%) and disability (15, 3%) in women.

**Figure 1:**
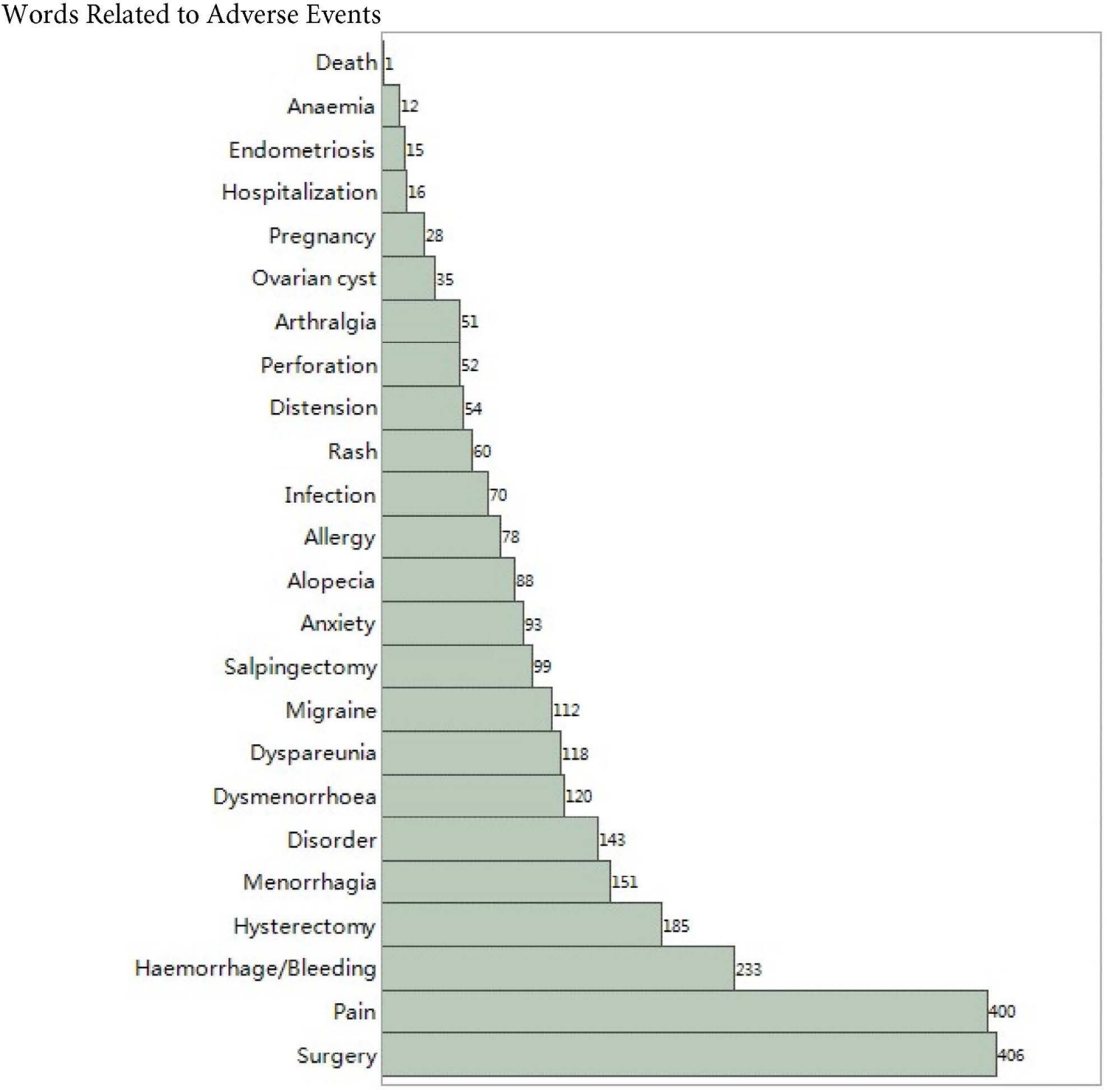
Frequency of 25 types of adverse events from 500 randomly selected cases.

Figure 1 shows the frequency of the top 25 words related to adverse events sorted from low to high. Surgery and pain accounted for more than 80% of the sample, followed by hemorrhage, hysterectomy, and menorrhagia. The proportion of hospitalization, endometriosis, anaemia, and death are less than 5%.

Pain was one of the adverse events that recurs in the collected reports. Quantitative analysis was performed on 12 types of pain, and the results are shown in Table 3. More than half of the reports mentioned the occurrence of pelvic pain (59%). Reports of abdominal pain exceeded 40%. A small number of patients (less than 5%) reported fibromyalgia, physical pain, or ovulation pain.

**Table 3:** Different type of pains associated with Essure medical device among 500 randomly selected cases in 2018.

Type of pain	*N* (weighted %)
Pelvic pain	295 (59.00)
Abdominal pain	201 (40.20)
Dysmenorrhea	120 (24.00)
Dyspareunia	118 (23.60)
Migraine	112 (22.40)
Headache	111 (22.20)
Back pain	108 (21.60)
Arthralgia	51 (10.20)
Virginal pain	32 (6.40)
Fibromyalgia	22 (4.40)
Physical pain	13 (2.60)
Ovulation pain	6 (1.20)

### 3.4. Thematic analysis of death-case sample of reports

Ten of all the cases reported death outcomes, including eight death cases of women, one death case of a fetus, and one death case of newborn (Supplementary Table S2). All ten cases were submitted by the manufacturer, and six were lawyer’s reports. Potential causes of death included deep vein thrombosis, intra-abdominal bleeding, uterine perforation (on the same day that the patient had Essure inserted), intestinal perforation (during the laparoscopy with bilateral salpingectomy was performed to remove metallic Essure remains), embolism, infection, and cervical cancer.

## 4. Discussion

These findings indicated several safety issues related to the Essure device. The reported complaints associated with the device during the ten months’ period include bleeding, pain, and heavy periods. Other complications include tubal or uterine perforation, intraperitoneal migration, unintended pregnancies, and device removal. Like with other obstetrics and gynecology devices, pain and bleeding are the most common adverse events among patients who had undergone Essure procedures (31–33).

According to the Medical Devices Amendment Act, Essure had been listed as a Class III, high risk medical device which requires a premarket approval (PMA) before it can be marketed in the U.S. (34–36). Compared to the process for most Class I devices, which are exempt from Premarket Notification 510(k), and Class II devices, which require Premarket Notification 510(k), the PMA is more complicated and involves clinical data (34, 35, 37). Based on nonrandomized, single-arm prospective clinical studies, the FDA conditionally granted Essure approval as a Class III medical device in November 2002 via an expedited review within the PMA process, which requires mandatory post-approval studies of five-year follow-up of participants in phase II and pivotal trials, as well as success rate for bilateral placement (34, 38, 39). From 2000 to 2015, 18 obstetrics and gynecology devices were introduced to the U.S. market through PMA (40). Of these, 42% were approved based on nonrandomized controlled trials, and three devices were ultimately withdrawn from the market. (Essure was not included in these three devices because it was withdrawn after this study was published) (40). Similarly, between 2000 and 2007, only 27% of studies supporting the PMAs for high risk cardiovascular devices were randomized (41). Considering the potential for adverse events, it is essential that medical devices, especially high-risk devices such as Essure, are subject to rigorous supervision.

The manufacturer submitted most of the reports of Essure during this period, which is consistent with a previous study of 22 years’ FDA MAUDE database that the major sources of the information are from the manufacturer (42). According to the Medical Device Reporting regulation (21 CFR Part 803), device manufacturers, importers, and user facilities are required to report device-related death or serious injury to the FDA, and user facilities should report adverse events to both manufacturers and the FDA. However, only three of 4,994 Essure reports were from user facilities, and less than 10% were from non-healthcare professionals and physicians. There were about 10% voluntary reports, while less than 1% were initially reported to the FDA. The reason of low voluntary reporting rate could be a lack of comprehensive structure which results in a time-consuming and inefficient reporting process (42).

### 4.1. Review of clinical studies

The lack of clinical data reported to clinicaltrials.gov indicates a significant problem regarding device performance, which is often shielded under trade secret provisions (43). As of February 2019, 23 clinical trials related to Essure medical devices can be found on the clinicaltrials.gov website (Supplementary Table S3). The manufacturer, Bayer, sponsored eleven trials, and 17% of all trials were conducted in the U.S. More than half of those trials have been completed, but only two have reported the results. One completed trial was sponsored by the University of New Mexico, Bayer, and the Society of Family Planning. It was a double blind, randomized study which focused on pain assessment and patient satisfaction and was verified in May 2016 (44). Another completed study was executed by Bayer in Canada and Mexico to assess and evaluate the effectiveness of the Essure System for Permanent Birth Control (ESS505). Researchers believed that as a modification to the commercially available ESS305 (previously Essure), the “new Essure” results in a high rate of both immediate-term and intermediate-term tubal occlusion without adverse events (45). To ensure the safety of medical devices and promote public trust and confidence in the medical device industry, it is essential to increase transparency and accountability in publishing clinical results.

Compared to the literature, the present study focused on the types of adverse events and the report itself. Results showed that even in the year when Essure was about to exit the U.S. market, the number of adverse events remained high, and most of the incidents in the collected reports occurred in the past 5 years. For women who still have an Essure implant, they should consult with their healthcare providers about existing or potential adverse events and appropriate solutions (46).

### 4.2. Limitations

It is recognized that adverse events are under-reported, can represent only the “tip of the iceberg”, and can be limited in narrative content. In this study, only the events and factors that were explicitly stated in free-text narratives were included. According to the FDA’s guideline, although medical device reports are a valuable source of information, this passive surveillance system has limitations including incomplete, inaccurate, unconfirmed, or biased data that may not be fully submitted. Under the current system, submitting a medical device report and the release of such information by the FDA is not regarded as a recognition of manufacturers or health care providers that made contributions to the event. In other words, this dataset suggests a potential association between these adverse events and the Essure device, but no causation can be established using this dataset, and it is impossible to calculate the rate of adverse events since the total number of people using the device is unknown. Also, the FDA MAUDE data does not include all known safety information, and some types of report information are protected from public disclosure under the Freedom of Information Act. In the report text, “(b)” (4) presents that it contains trade secret or confidential commercial information and something about the maker cannot be found. Similarly, a patient’s age is replaced by “(b)” (6) because it is considered personal or medical information. The occupation category “Other” in the Reporter Occupation Code is not clearly defined in the dataset, and therefore, we do not have information about what it specifically refers to (26). Some adverse events, especially serious adverse events, could be associated with infections and complications during medical interventions. Some reports also mentioned patients’ medical history, such as systemic lupus erythematosus and Sjogren’s syndrome. Another limitation is the potential for duplication of reports, since a report for the same patient may be submitted by multiple entities. Similarly, there could be a potentially inflated percentage of death and other adverse outcomes due to the size of the random sample, which could result in an overestimation of the risk associated with the device.

### 4.3. Recommendations

The quality of adverse event reports needs to be improved to minimize the bias from incomplete and subjective data. More health professionals need to fill out voluntary report form, such as using the MedWatch system, to provide more safety information about medical devices. In addition, effective medical device management necessitates prompt investigation of complaints, rapid dissemination of relevant information to both regulators and users, and implementation of appropriate corrective actions to identify potential risks early and minimize harm to patients. In the future, more meaningful pre- and post-marketing surveillance and regulation are warranted in the medical device market. It is also important for FDA and the academic institutions to keep the focus on the study research of the “New Essure” and raise awareness among physicians and patients.

## 5. Conclusion

In light of these safety issues of medical devices, more meaningful pre- and post-marketing surveillance and regulation are warranted in the medical device market. Based on the assessment of existing reports and the pre- and post-market surveillance, the cooperation between patients, doctors, lawyers, the FDA, and academic institutions is required to issue a safety alert at an earlier stage with a more proactive safety surveillance system and stricter regulation of the class III medical devices, especially the products like Essure or the ‘new Essure’. Safety adverse reporting systems of medical devices need to be further improved for public understanding and scientific research.

## Data Availability

Publicly available datasets were analyzed in this study. This data can be found here: https://www.accessdata.fda.gov/scripts/cdrh/cfdocs/cfmaude/search.cfm.
