# Supplementary material for: Safety reporting of Essure medical device: a qualitative and quantitative assessment on the FDA manufacturer and user facility device experience database in 2018
Source: Front Reprod Health. 2023 Jul 13;5:1172927. doi: 10.3389/frph.2023.1172927 (PMC10374426; doi:10.3389/frph.2023.1172927)
Supplement: Supplementary file 1 [file Table1.docx]

Supplementary Material

Safety Reporting of Essure Medical Device: A Qualitative and Quantitative Assessment on the FDA Manufacturer and User Facility Device Experience Database in 2018

Chenyu Zou^1*^, Brandy Davis^1^, Patricia R. Wigle^2^, Ana L. Hincapie^2^, Jeff Jianfei Guo^2^

^1^Department of Health Outcomes Research and Policy, Harrison College of Pharmacy, Auburn University, Auburn, AL, United States

^2^Division of Pharmacy Practice & Administrative Sciences, The James L. Winkle College of Pharmacy, University of Cincinnati, Cincinnati, OH, United States

*** Correspondence:**Chenyu Zou, MS, Ph.D. Candidate
czz0063@auburn.edu

# Supplementary Tables

**Supplementary Table 1.** Reported Device Problems Associated with Essure Medical Device among 500 Randomly Selected Cases in 2018

| **Device Problem Reported** | **N (%)** |
| --- | --- |
| Adverse Event Without Identified Device or Use Problem | 187 (37.40) |
| Activation Failure Including Expansion Failures | 1 (0.2) |
| Appropriate Term/Code Not Available | 1 (0.2) |
| Biocompatibility | 24 (4.80) |
| Break | 13 (2.60) |
| Expulsion | 2 (0.40) |
| Device Appears to Trigger Rejection | 6 (1.20) |
| Failure to Advance | 1 (0.20) |
| Insufficient Information | 23 (4.60) |
| **Total** | **258** |

**Supplementary Table 2.** Ten Reported Death Cases Associated with Essure Medical Device Identified from the FDA MAUDE Database in 2018

| Death Report No.1 | |
| --- | --- |
| **Report Number** | 2951250-2018-00373 |
| **Event Date** | 07/03/2011 |
| **Event Type** | Death |
| **Date Received** | 01/15/2018 |
| **Device Problem** | Device dislodged or dislocated; adverse event without identified device or use problem; insufficient information |
| **Date of death** | 2016 |
| **Report Source** | Manufacturer |
| **Adverse Event** | Patient’s death |
| **Treatment** | Amitriptyline, Hydrocodone, Iron, Levothyroxine, Nexium Sandoz, Phenergan, Prevacid, Promethazine, Xanax, Zoloft |
| **Cause of Death** | Deep vein thrombosis |
|  | |
| Death Report No.2 | |
| **Report Number** | 2951250-2018-00557 |
| **Event Date** | N/A |
| **Event Type** | Death |
| **Date Received** | 02/09/2018 |
| **Device Problem** | Adverse Event Without Identified Device or Use Problem |
| **Date of death** | Unknown |
| **Report Source** | Manufacturer (This spontaneous case was posted on a Facebook page and refers to an unspecified female consumer) |
| **Adverse Event** | Patient’s death |
| **Treatment** | Not mentioned |
| **Cause of Death** | Not mentioned |
| Death Report No.3 | |
| **Report Number** | 2951250-2018-00624 |
| **Event Date** | N/A |
| **Event Type** | Death |
| **Date Received** | 2018/02/13 |
| **Device Problem** | N/A |
| **Date of death** | 2015 |
| **Report Source** | Manufacturer (This spontaneous case was reported by a lawyer) |
| **Adverse Event** | Intra-abdominal haemorrhage  Seriousness criteria death, medically significant and intervention required; Device dislocation; seriousness criteria medically significant and intervention required |
|  | Patient’s death |
| **Treatment** | Surgery (Hysterectomy) |
| **Cause of Death** | Intra-abdominal bleeding |
| Death Report No.4 | |
| **Report Number** | 2951250-2018-01279 |
| **Event Date** | 2010/01/14 |
| **Event Type** | Death |
| **Date Received** | 2018/03/27 |
| **Device Problem** | N/A |
| **Date of death** | 2017 |
| **Report Source** | Manufacturer (This spontaneous case was reported by a lawyer) |
| **Adverse Event** | Uterine perforation (seriousness criterion medically significant) with pelvic pain and abdominal pain lower. Genital haemorrhage (seriousness criterion medically significant) |
|  | Menorrhagia (increased bleeding during menstruation) |
|  | Vaginal haemorrhage, vaginal discharge and vaginal infection |
|  | Cstitis (infection bladder) and bladder disorder with bladder pain; Urinary tract infection and urinary tract disorder |
|  | Dysmenorrhoea, emotional distress, migraine, rash, back pain, fatigue, nervous system disorder (neurological conditions or problems), renal pain, tooth disorder, weight decrease |
|  | Patient’s death |
| **Treatment** | Not mentioned |
| **Cause of Death** | Incident due to a uterine perforation during Essure insertion procedure. |
| Death Report No.5 | |
| **Report Number** | 2951250-2018-02103 |
| **Event Date** | N/A |
| **Event Type** | Death |
| **Date Received** | 2018/05/08 |
| **Device Problem** | Break |
| **Date of death** | Undisclosed |
| **Report Source** | Manufacturer |
| **Adverse Event** | Procedural intestinal perforation (seriousness criterion death) |
|  | Pain (seriousness criteria medically significant and intervention required), Device breakage (seriousness criteria medically significant and intervention required); Complication of device removal (metallic Essure remains from first surgery) |
|  | Malaise (seriousness criterion medically significant) |
| **Treatment** | Surgery (procedure to remove Essure (first time)) |
|  | Surgery (laparoscopy/bilateral total salpingectomy and underwent to a bilateral total salpingectomy to remove metallic Essure remains (second time) |
| **Cause of Death** | A severe intestinal perforation during the second surgery |
| Death Report No.6 | |
| **Report Number** | 2951250-2018-02435 |
| **Event Date** | N/A |
| **Event Type** | Death |
| **Date Received** | 06/01/2018 |
| **Device Problem** | N/A |
| **Date of death** | Not mentioned |
| **Report Source** | Manufacturer (This spontaneous case was reported by a lawyer) |
| **Adverse Event** | Medical device removal (seriousness criteria medically significant and intervention required) |
|  | Mbolism (seriousness criterion death) |
|  | Malaise |
| **Treatment** | Surgery (Hysterectomy) |
| **Cause of Death** | Embolism |
| Death Report No.7 | |
| **Report Number** | 2951250-2018-02815 |
| **Event Date** | N/A |
| **Event Type** | Death |
| **Date Received** | 06/26/2018 |
| **Device Problem** | N/A |
| **Date of death** | Not mentioned |
| **Report Source** | Manufacturer (This spontaneous case was reported by a lawyer) |
| **Adverse Event** | Medical device removal (seriousness criteria medically significant and intervention required) |
|  | Mbolism (seriousness criterion death) |
|  | Malaise |
| **Treatment** | Surgery (Hysterectomy) |
| **Cause of Death** | Embolism |
| Death Report No.8 | |
| **Report Number** | 2951250-2018-02989 |
| **Event Date** | N/A |
| **Event Type** | Death |
| **Date Received** | 07/06/2018 |
| **Device Problem** | N/A |
| **Date of death** | Unknown |
| **Report Source** | Manufacturer (This spontaneous case was reported by a lawyer) |
| **Adverse Event** | Fatal infection (baby died shortly after birth due to an infection due to Essure) |
| **Treatment** | Not mentioned |
| **Cause of Death** | Infection |
| Death Report No.9 | |
| **Report Number** | 2951250-2018-03413 |
| **Event Date** | N/A |
| **Event Type** | Death |
| **Date Received** | 08/09/2018 |
| **Device Problem** | N/A |
| **Date of death** | 2017 |
| **Report Source** | Manufacturer (This spontaneous case was reported by a lawyer) |
| **Adverse Event** | Pelvic pain (seriousness criteria medically significant and intervention required) |
|  | Genital haemorrhage (seriousness criterion medically significant) |
|  | Cervix carcinoma (seriousness criterion death) |
|  | Salpingitis (seriousness criteria medically significant and intervention required) |
|  | Fallopian tube perforation (seriousness criteria medically significant and intervention required) |
| **Treatment** | Chemotherapy and surgery (left salpingo-oophorectomy and Essure was removed) |
| **Cause of Death** | Cervical cancer |
| Death Report No.10 | |
| **Report Number** | 2951250-2018-03728 |
| **Event Date** | N/A |
| **Event Type** | Death |
| **Date Received** | 08/27/2018 |
| **Device Problem** | N/A |
| **Date of death** | Not mentioned |
| **Report Source** | Manufacturer |
| **Adverse Event** | Device dislocation (seriousness criteria medically significant and intervention required) |
|  | Fetal death (seriousness criterion medically significant) |
|  | Autoimmune disorder (seriousness criterion medically significant) |
|  | Pregnancy with contraceptive device (seriousness criterion medically significant) |
| **Treatment** | Surgery (body parts ripped and Essure was removed) |
| **Cause of Death** | Not mentioned |

**Supplementary Table 3.** Analysis of Available Clinical Trials Related to Essure Medical Devices Found on the Clinicaltrials.gov Website

| **Characteristic** | | **N. (%)** |
| --- | --- | --- |
| Status | Not yet recruiting | 1 (4.35) |
|  | Recruiting | 2 (8.70) |
|  | Enrolling by invitation | 1 (4.35) |
|  | Active, not recruiting | 4 (17.39) |
|  | Terminated | 1 (4.35) |
|  | Completed | 12 (52.17) |
|  | Withdrawn | 1 (4.35) |
| Eligibility Criteria | Accepts Healthy Volunteers | 12 (52.17) |
|  | Not Accepts Healthy Volunteers | 11 (47.83) |
| Study Type | Interventional (Clinical Trial) | 12 (52.17) |
|  | Observational | 11 (47.83) |
| Study Results | With Results | 2 (8.70) |
|  | Without Results | 21 (91.30) |
| Study Phase | Phase 1 and Phase 2 | 1 (4.35) |
|  | Phase 3 | 1 (4.35) |
|  | Phase 4 | 1 (4.35) |
|  | Not Applicable | 20 (86.96) |
| Funder Type | Industry | 13 (56.52) |
|  | Individuals, Universities, Organizations | 10 (43.48) |
| Location | Europe | 8 (34.78) |
|  | Canada | 3 (10.71) |
|  | Mexico | 1 (4.35) |
|  | United States | 16 (69.57) |
